# Arthroscopic Technique for Repairing Medial Meniscus Posterior Root Using a Handmade Suspensory Fixation System

**DOI:** 10.1016/j.eats.2024.103422

**Published:** 2025-01-11

**Authors:** Harun Altınayak, Yavuz Selim Karatekin, Ramazan Akmeşe

**Affiliations:** a Department of Orthopaedics and Traumatology, Health Sciences University Samsun Training and Research Hospital, Samsun, Turkey; b Department of Orthopaedics and Traumatology, Cankaya Hospital, Ankara, Turkey

## Abstract

This report describes an arthroscopic technique that utilizes a handmade suspensory fixation system designed to optimize suture tension and provide stable fixation in meniscal root repair. The technique allows for simultaneous and adjustable tensioning of all sutures, aiming to enhance stability and improve clinical outcomes. While further validation through clinical studies is required, this technique has the potential to increase the success of medial meniscus posterior root tear repairs and improve long-term functional outcomes for patients.

The integrity of the posterior meniscal root is essential for maintaining proper knee biomechanics.^1^^,^^2^ A medial meniscus posterior root tear (MMPRT) disrupts knee kinematics, resulting in a substantial increase in tibiofemoral contact pressure.^1^ Recent systematic reviews and meta-analyses have shown that surgical repair of the MMPRT is superior to nonsurgical treatments or meniscectomy in improving patient-reported outcomes and mitigating the progression of osteoarthritis.^3^, ^4^, ^5^

Numerous arthroscopic techniques for meniscal root repair have been described, with the transtibial pull-out repair being the most prevalent.^6^, ^7^, ^8^ Extensive research has investigated the biomechanical durability and clinical outcomes associated with various suture techniques employed in these procedures.^9^^,^^10^ Additionally, the impact of different suture materials and various suture passers on meniscal root repair has been explored in both biomechanical and clinical studies.^11^, ^12^, ^13^ Ongoing advancements aim to improve repair outcomes through the development of new techniques and devices.^14^, ^15^, ^16^ Despite these advancements, achieving optimal tension in the meniscal root and preventing gapping at the repair site remain challenging. A biomechanical study has shown that a 3-mm medial displacement of the meniscal root reduces the tensile force from femorotibial load by 49% to 68%.^17^^,^^18^ Ensuring appropriate tension during transtibial pull-out repair is crucial to prevent displacement after the procedure.

In this context, our developed “handmade suspensory fixation system” is designed to address secondary laxity by enabling simultaneous tensioning of all sutures passing through the meniscal root and allowing for retensioning of the repaired root if needed. This technique aims to enhance the overall stability and efficacy of the repair by maintaining the desired tension in the button implant fixation across varying degrees of knee flexion.

## Surgical Technique

### Patient Positioning and Diagnostic Arthroscopy

**Fig 1:**
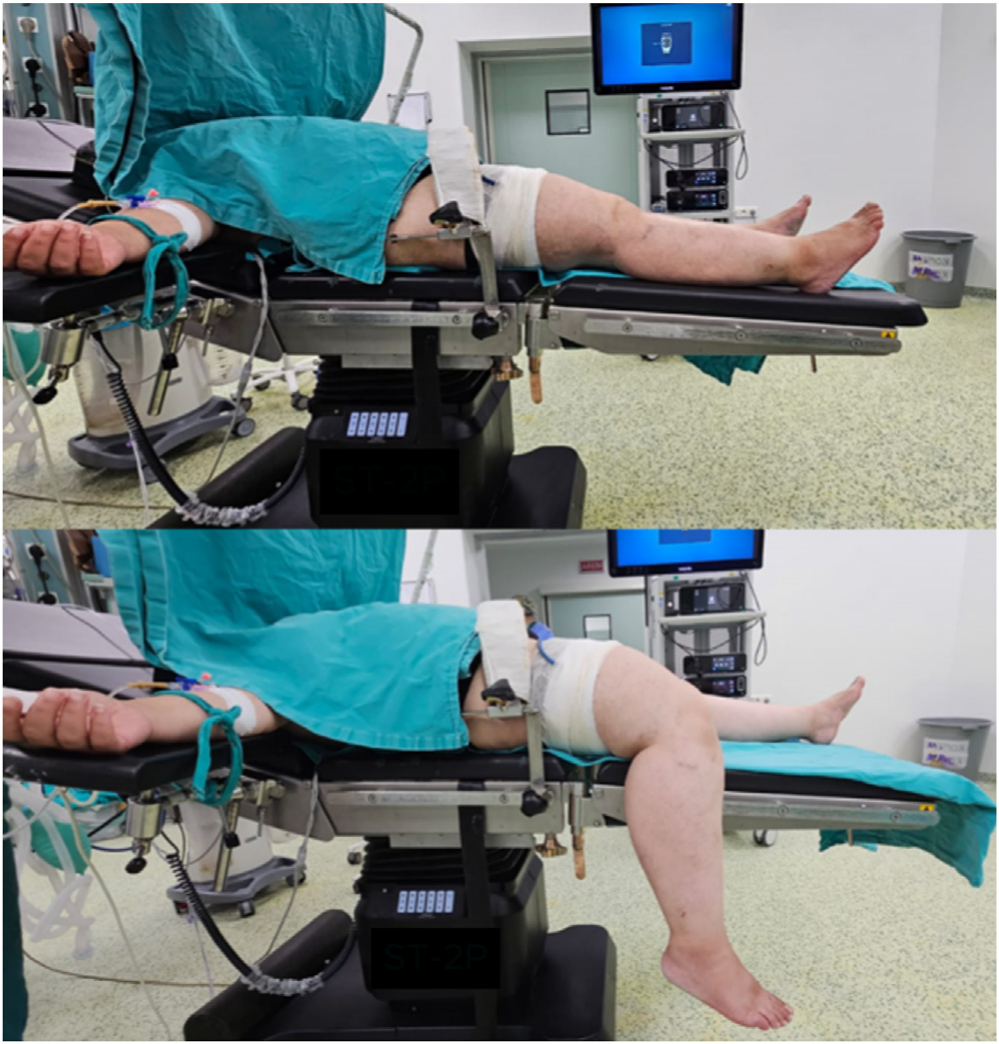
Patient positioning on the surgical table.

The patient is positioned supine with the knee flexed and aligned to the side of the operating table. A side post is used to position the knee in valgus for better visualization of the medial compartment (Fig 1). The arthroscopy is performed with a 30° scope (Stryker) using standard anterolateral (AL) and anteromedial (AM) portals. Medial compartment release may be necessary to improve visualization and prevent cartilage damage^19^ (Table 1). The posterior meniscal root tear is assessed and classified according to the LaPrade system for the medial meniscus.^20^

**Table 1:** Pearls and Pitfalls of the Technique for Repairing the Medial Meniscus Posterior Root Using a Handmade Suspensory Fixation System

Pearls	Pitfalls
Verify the correct indication and that the patient can comply with postoperative guidelines.	To prevent the sutures from damaging the meniscus tissue during sliding, it is recommended to create the closed loop in the sutures near the tibial tunnel.
Consider drilling the tunnel from the anterolateral tibia to have a better pull direction for the medial meniscus posterior root.	Avoid overtensioning the meniscus, as this can cause the meniscus to tear.
Use a cannula in the anteromedial portal for suture management.	Range the knee through range of motion and place it is varus stress to check if final tension is preserved, and otherwise ensure retensioning of the construct.
Ensure an anatomic tibial tunnel position is created.	
Use a needle pie-crusting technique to release some part of the medial collateral ligament in the tight medial compartment knee.	
Ensure that the initial loop knots created for the suspensory system are positioned close to the knot forming the closed loop.	
Ensure that the gaps in the simple loop sutures are eliminated before starting to tension the suspensory system.	

### Preparation of the Medial Meniscus Posterior Root Tear

To improve the view of the posterior root footprint, a portion of the medial tibial eminence and the posterior cruciate ligament synovium is cleared. The tear edges are debrided, and the bone bed is decorticated with a curette to promote healing (Video 1). In chronic extrusion cases, peripheral fixation and release of the meniscotibial ligament are recommended to allow mobilization into the bone bed.

### Passing of the Medial Meniscus Posterior Root Sutures

**Fig 2:**
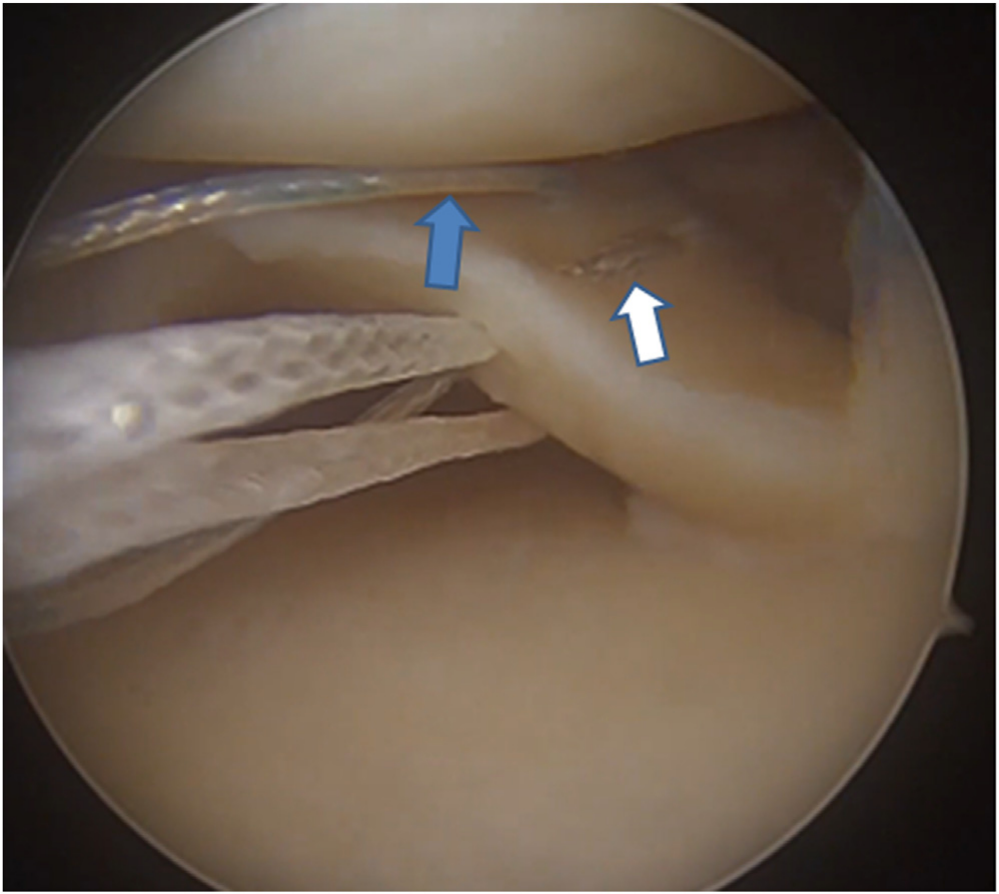
The patient is in the supine position. The posterior root of the medial meniscus of the right knee is being visualized through the anterolateral portal. Insertion of sutures using an arthroscopic suture passer in accordance with the modified Mason-Allen suture technique for the medial meniscus posterior root. The blue arrow indicates the blue-white thread forming the vertical suture component of the modified Mason-Allen suture technique. The white arrow indicates the white thread forming the horizontal suture component of the modified Mason-Allen suture technique.

Using an AL view, a 5-mm cannula (Orthomed) is inserted into the AM portal (Table 1). Two nonabsorbable sutures (2/0 Klotho fiber; Orthomed) are passed through the root using the First Pass Mini device (Smith & Nephew) with a modified Mason-Allen technique (Fig 2, Video 1). A tibial guide is placed at the footprint, and a 2-cm incision is made anterolaterally on the tibia (Table 1). A Kirschner wire is inserted and verified arthroscopically, and a 4.5-mm drill is used to create a tibial tunnel. A No. 1 PDS suture is passed through the tunnel and retrieved through the AM portal. The root sutures are tied to the carrier suture and pulled through the tibia.

### Creation of the Handmade Suspensory Fixation System and Completion of the Medial Meniscus Posterior Root Fixation

**Fig 3:**
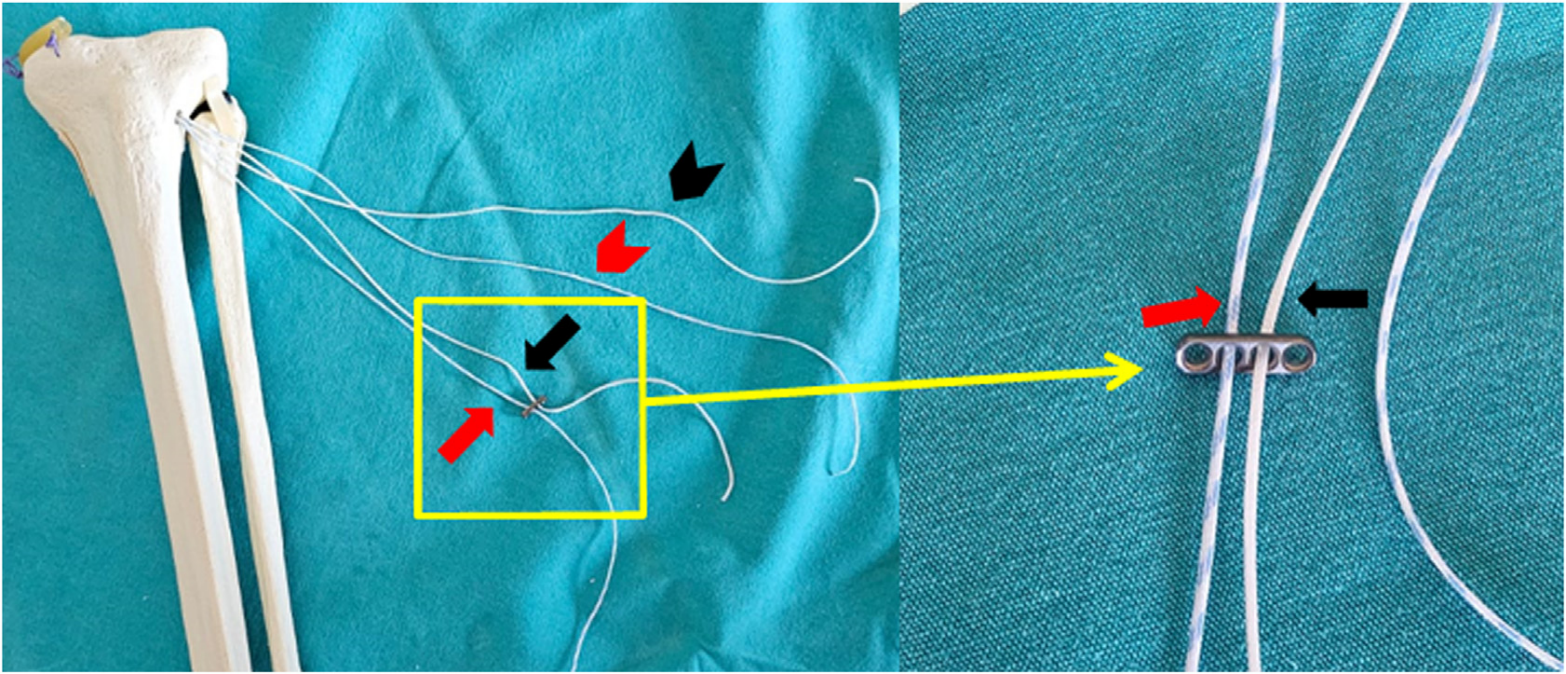
Passage of the ends of sutures of different colors through the contiguous holes located at the center of the button implant. The red arrow indicates the blue-white suture passing through the button implant. The black arrow indicates the white suture passing through the button implant. The red arrowhead indicates the other free end of the blue-white suture. The black arrowhead indicates the other free end of the white suture.

**Fig 4:**
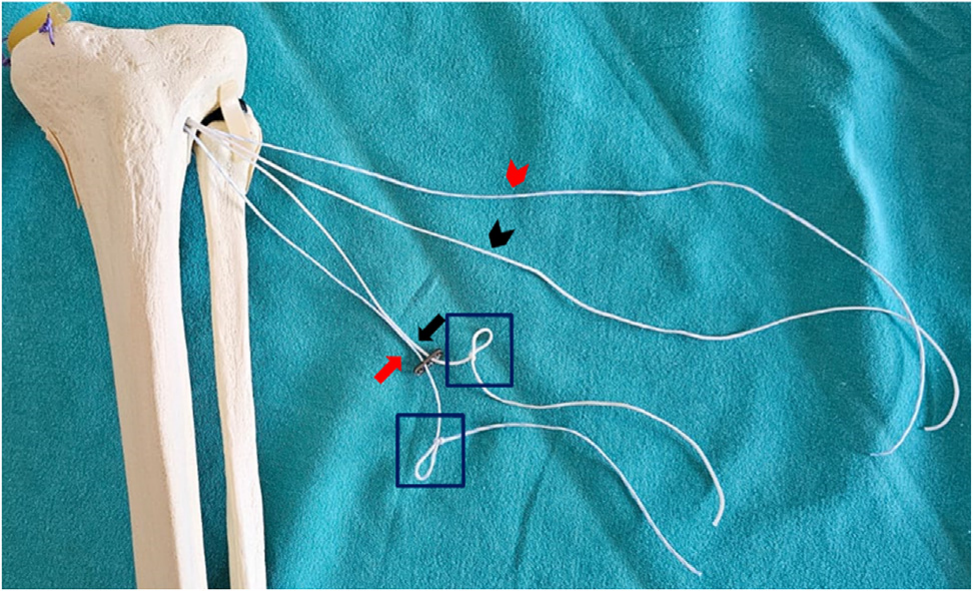
Formation of loop sutures in each suture threaded through the holes of the button implant. The red arrow indicates the blue-white suture passing through the button implant and forming a simple loop. The black arrow indicates the white suture passing through the button implant and forming a simple loop. The red arrowhead indicates the other free end of the blue-white suture. The black arrowhead indicates the other free end of the white suture. Inside the rectangle indicates the simple loops formed.

At this stage, the different-colored sutures brought to the AL region of the tibia are passed through the adjacent holes located in the center of the button implant (Orthomed) (Fig 3). As the sutures are threaded through the holes of the button implant, the first step of creating loops begins. Each suture end is twisted and tied securely to form a simple loop. These loop knots are carefully tied to create a stable structure and are optimized to resist both tensile forces and cyclic loading (Fig 4, Table 1).

**Fig 5:**
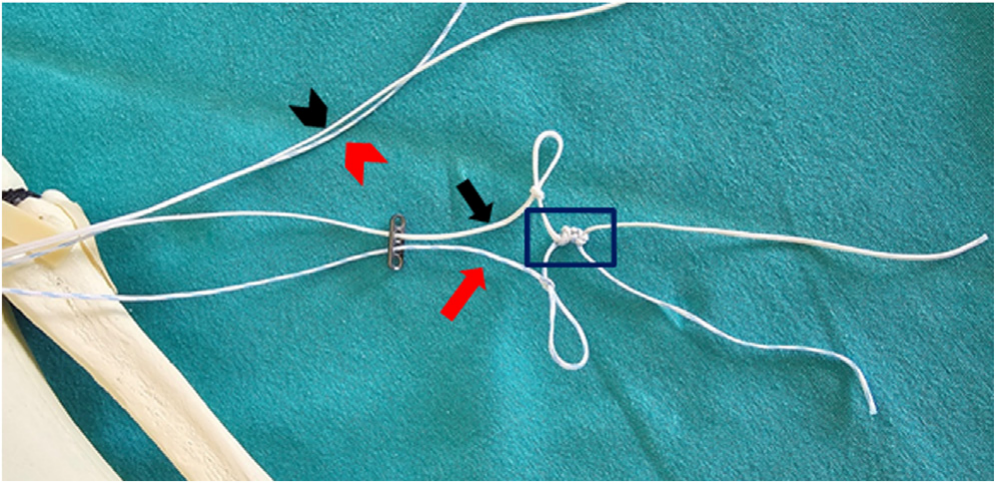
Formation of a closed loop by tightly securing the ends of 2 sutures used to create loop sutures. The red arrow indicates the blue-white suture passing through the button implant and forming a closed loop. The black arrow indicates the white suture passing through the button implant and forming a closed loop. The red arrowhead indicates the other free end of the blue-white suture. The black arrowhead indicates the other free end of the white suture. Inside the rectangle shows the knotted ends of sutures in different colors tied together to form a closed loop.

Subsequently, these 2 ends are tightly tied together, forming a closed loop (Fig 5, Table 1). This loop is designed to securely fix the button implant and forms the foundation of the suspensory system used for meniscus root fixation. The closed loop structure adds additional stability to the suspensory fixation system, providing resistance against loosening that might occur during the repair process.

**Fig 6:**
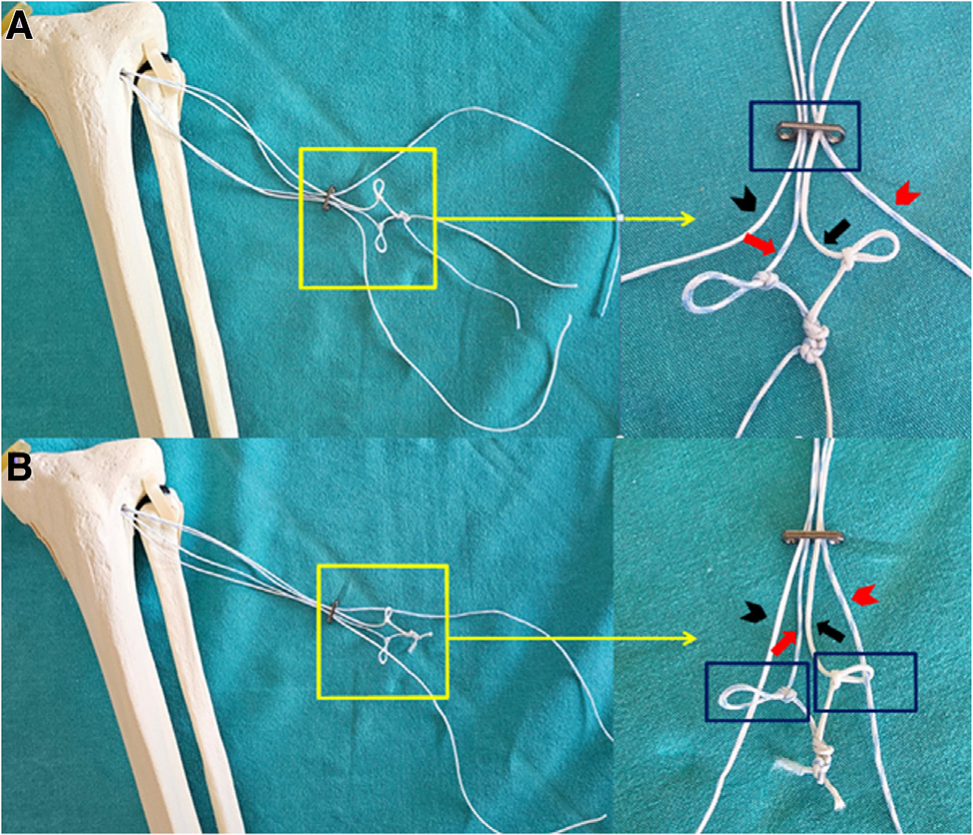
Passage of the free ends of the sutures through the button implant hole corresponding to the contrasting suture color (A) and through its suture loop (B), creating a cross-linking structure. The red arrow indicates the blue-white suture forming a cross-linking structure. The black arrow indicates the white suture forming a cross-linking structure. The red arrowhead indicates the blue-white suture passing through the button implant hole (A) and the suture loop (B), forming one of the pulling arms of the handmade suspensory system. The black arrowhead indicates the white suture passing through the button implant hole (A) and the suture loop (B), forming one of the pulling arms of the handmade suspensory system. Inside the rectangle indicates the passage of the other free ends of the sutures through the button implant (A) and through the suture loop (B).

**Fig 7:**
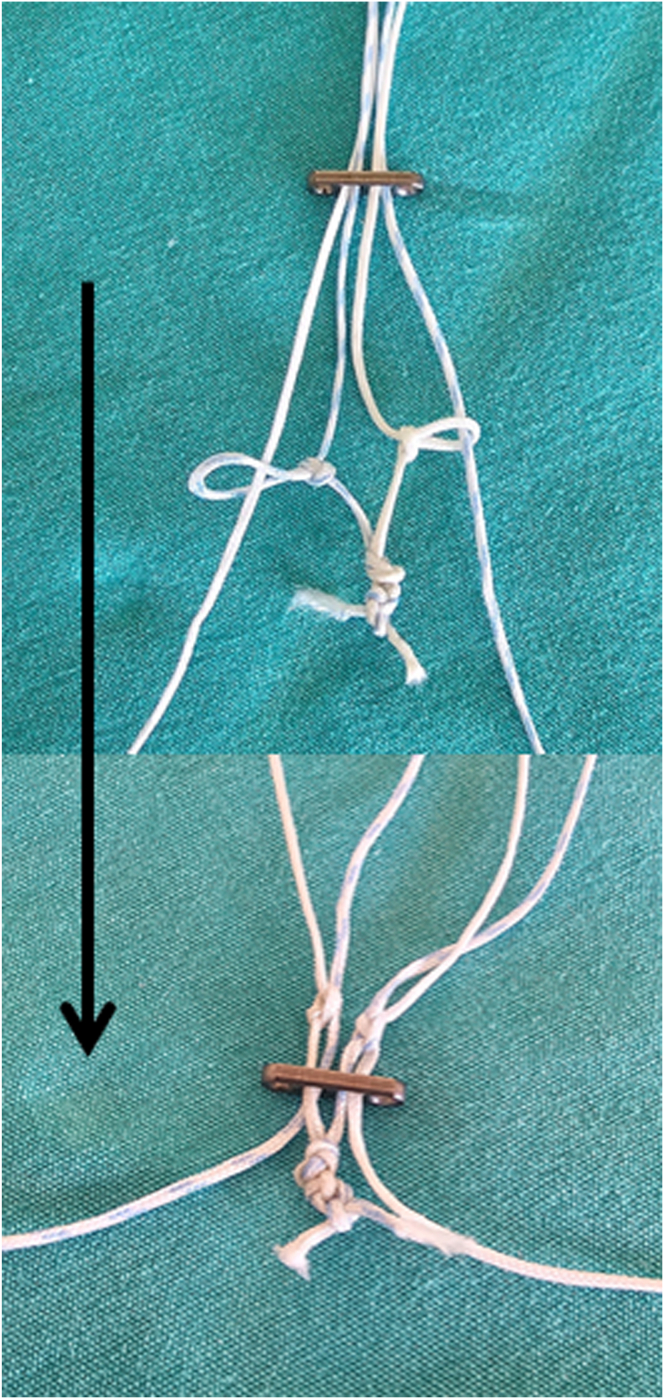
The button implant is passed distal to the loop sutures.

Then, the other ends of each suture are passed through the hole in the button implant where the opposing colored suture has passed and through the loop of this suture, creating a cross-linking structure (Fig 6). Subsequently, the button implant is passed distal to the loop sutures (Fig 7). Additionally, at this step, the gaps in the simple loop sutures are also eliminated (Table 1). These steps are critical for establishing a secure suspensory fixation system, ensuring that each suture is both securely fixed in place and integrated with the others to form a complete structure. By bringing the sutures together in this way, the button implant is optimally stabilized, and a uniform load distribution is ensured during the fixation of the meniscus root.

Next, the free ends of the sutures are manually pulled, and under arthroscopic visualization, the desired tension of the posterior meniscal root is achieved (Video 1, Table 1). During this process, the adequacy of the suture tension and the firm fixation of the posterior meniscus root to the bone are carefully observed using the arthroscope. If necessary, fine adjustments can be made to positioning the button implant and the tension settings. Another point to be mindful of at this stage is to avoid damaging the meniscal tissue and to ensure that the sutures do not excessively tighten, which could cut through the meniscal tissue (Table 1). After the system is successfully set up and the tension adjustments are made, the meniscal root is securely fixed to the bone without any gap.

## Discussion

The recognition and treatment of MMPRTs have become significant research areas due to increased awareness.^5^^,^^21^, ^22^, ^23^ Techniques for MMPRT repair include transtibial pull-out, anchor suture repair, gracilis autograft, and all-inside suture repair.^24^, ^25^, ^26^, ^27^ The transosseous method is the most commonly used, although biomechanical studies have shown it can result in a gap of up to 2 to 3 mm, potentially leading to meniscus extrusion.^28^, ^29^, ^30^, ^31^ While transtibial techniques are considered the gold standard, their limitations have prompted the development of innovative solutions.^14^, ^15^, ^16^

The success of MMPRT repair depends on both primary fixation strength and resistance to cyclic loading. Our approach, based on the modified Mason-Allen suture technique, allows simultaneous adjustment of all suture tensions. This technique aims to enhance repair effectiveness by enabling retensioning and ensuring more uniform pressure distribution over the meniscus root tissue.^32^^,^^33^

It is hypothesized that MMPRT repairs with a higher resistance to cyclic displacement may improve healing outcomes.^34^^,^^35^ Our technique also permits the knee to rotate through a range of motion and allows multiple retensioning of the repaired root to ensure solid fixation without gaps.

A recent biomechanical study compared traditional fixed transtibial pull-out repair techniques with a new adjustable device providing rip-stop suture repair and intratunnel soft anchor fixation. The findings showed that adjustable intratunnel soft anchor fixation offered higher initial repair load and resistance to displacement compared to fixed repairs, suggesting biomechanical advantages.^36^ However, adjustable sutures require separate tensioning, which can complicate equal tensioning. In contrast, our technique offers simultaneous equal-force tensioning.

Our method, based on the modified Mason-Allen suture repair, provides adjustable tension across all sutures, which may facilitate better meniscus healing and superior biomechanical performance.^37^^,^^38^ Therefore, we prefer this technique, although other self-sliding suture methods could also be used.

Limitations include the need for further validation of the biomechanical performance and clinical follow-up to assess effectiveness. Additionally, fixation strength might exceed meniscal tissue resilience, and excessive tension could lead to suture cut-through. Thus, overtensioning should be avoided.

In conclusion, our technique offers significant biomechanical advantages for MMPRT repair and could improve repair success and long-term functional outcomes for patients.

## Disclosures

All authors (H.A., Y.S.K., R.A.) declare that they have no known competing financial interests or personal relationships that could have appeared to influence the work reported in this paper.

## Supplementary Data

Video 1 Step-by-step surgical technique for arthroscopic transtibial pull-out fixation of a medial meniscus posterior root tear using a handmade suspensory system. The posterior medial meniscus repair is shown on a right knee, viewed through the anterolateral portal, with the patient in the supine position and the knee flexed at 90°.
